# Prevalence of atherosclerotic cardiovascular disease and subsequent major adverse cardiovascular events in Alberta, Canada: A real‐world evidence study

**DOI:** 10.1002/clc.23732

**Published:** 2021-09-29

**Authors:** Guanmin Chen, Megan S. Farris, Tara Cowling, Lionel Pinto, Raina M. Rogoza, Erin MacKinnon, Salimah Champsi, Todd J. Anderson

**Affiliations:** ^1^ Medlior Health Outcomes Research Ltd. Calgary Alberta Canada; ^2^ Libin Cardiovascular Institute, University of Calgary Calgary Alberta Canada; ^3^ Amgen Inc. Thousand Oaks California USA; ^4^ Amgen Canada Inc. Mississauga Ontario Canada

**Keywords:** atherosclerotic cardiovascular disease, acute myocardial infarction, major adverse cardiovascular events, prevalence, real‐world evidence

## Abstract

**Background:**

Atherosclerotic cardiovascular disease (ASCVD) is a leading cause of morbidity and mortality worldwide. Data from Canadian populations regarding the burden of ASCVD are limited. Therefore, we describe the 5‐year period prevalence of ASCVD and subsequent major adverse cardiovascular event (MACE) outcomes among patients with ASCVD in Alberta, Canada.

**Methods:**

A retrospective, observational study was conducted by linking provincial health services data, vital statistics, and pharmaceutical dispenses data. Five‐year period prevalence of clinical ASCVD was captured between 2011 and 2016, and a cohort of adult patients with an initial clinical ASCVD event were identified between 2012 and 2016. One‐year incidence rates (IRs) of subsequent MACE outcomes were calculated as composite and individual measures. A subgroup of patients with acute myocardial infarction (AMI) as their index event was examined.

**Results:**

There were 198 573 patients (mean [standard deviation] age: 63.9 [15.6] years; 56.6% males) identified with clinical ASCVD between 2012 and 2016. Overall, the 5‐year period prevalence of ASCVD in Alberta was 89.9 per 1000 persons and the 1‐year IR for a primary MACE outcome was 6.15 (95% confidence interval [CI]: 6.03–6.26) per 100 person‐years. Among the ASCVD cohort, 9465 had an AMI as their index event and the IR for a primary MACE outcome was 14.30 (95% CI: 13.45–15.20) per 100 person‐years.

**Conclusions:**

This study found that the prevalence of ASCVD and the rate of subsequent MACE outcomes 1 year following the initial ASCVD event are substantial, particularly among patients with an AMI. Secondary prevention strategies aimed at lowering this risk are needed for patients with ASCVD.

## 1 INTRODUCTION

ASCVD continues to be a leading cause of morbidity and mortality worldwide.^1^ A global mortality rate of over 17.9 million people is associated with cardiovascular (CV) diseases annually, accounting for 31% of overall deaths per year.^2^ Similarly, in Canada, not only is ASCVD the second leading cause of death following cancer, but the 10‐year risk of a CV event was 8.9%.^3^, ^4^ In addition to the considerable impact on patient health, the global cost of ASCVD in 2010 was estimated to be $863 billion USD.^1^ Although estimates of the financial burden of ASCVD across Canada are lacking, a recent study conducted in Ontario, Canada reported the cost associated with newly diagnosed ASCVD was $66.6 billion CAD between 2005 and 2016,^5^ further highlighting the burden of ASCVD on the Canadian healthcare system.

Recent trends also suggest that mortality rates, among those with ASCVD, are rising due to an increasing prevalence of cardiometabolic risk factors, such as diabetes and obesity,^6^, ^7^ and the risk of recurrent CV events.^8^ Pre‐existing ASCVD has been shown to predict recurrent CV events in the same or different arteries or arterial beds.^9^ Several trials investigating patients with already established acute coronary syndromes (ACS) found that the rate of subsequent CV events over 8–17 months was 7.5%–19.9%.^10^, ^11^, ^12^, ^13^, ^14^, ^15^ Similarly, when pooling individual patient‐level data from four post‐ACS trials of 46 694 patients with a median follow‐up of 358 days, the rate of myocardial infarction (MI), stroke, or CV death combined was 9.2%, with MI events being the most reported event at 5.8%.^16^ In addition, this study reported that almost one‐third of patients with a first event, then had a subsequent event; a subsequent MI was the most common secondary event among those with an initial MI.^16^ A Canadian study reported that acute MI (AMI) had one of the highest rates of hospitalization, especially among men,^17^ relative to other CV events. Although preventative strategies, such as lipid‐lowering therapies (LLT) and lifestyle modifications are well‐established and recommended to improve patient outcomes,^18^ there remains a high residual risk for recurrent MACE outcomes, and in particular, more severe ASCVD conditions, such as AMI.^16^, ^17^, ^19^

Although previous publications worldwide have examined the burden of ASCVD, there is limited Canadian evidence evaluating the burden of ASCVD and subsequent MACE outcomes, including studies with real‐world data.^20^ In particular, some evidence has shown that patients outside of a clinical trial setting (i.e., real‐world studies) experience more severe outcomes.^21^, ^22^ Therefore, the aim of the current study was to confirm the findings in previous literature that subsequent MACE outcomes among patients with clinical ASCVD are substantial, utilizing real‐world population‐based data from the Albertan health system. The objectives of the study were to estimate the 5‐year period prevalence of ASCVD in Alberta, to describe the 1‐year incidence of subsequent MACE outcomes after an ASCVD event, and to examine a subgroup of patients with AMI as their index ASCVD event.

## 2 MATERIALS AND METHODS

### 2.1 Study design

A retrospective observational study was conducted using province‐wide health administrative data from Alberta, Canada.

### 2.2 Data sources

The following datasets were used in this study: (1) Discharge Abstract Database (DAD), including inpatient hospital diagnostic information; (2) National Ambulatory Care Reporting System (NACRS), including facility‐based ambulatory care information on services and diagnoses (e.g., emergency department visits); (3) Practitioner Claims, containing fee‐for‐service claims from practitioners and other insured health services (e.g., specialists); (4) Population Registry, including demographic and geographic information for all individuals who are registered with the Alberta Health Care Insurance Plan (i.e., Albertan residents); (5) Pharmaceutical Information Network (PIN), including medication type, dosage, and days supply; and (6) Vital Statistics, including date and cause of death.

The study complies with the Declaration of Helsinki and was approved by the Health Research Ethics Board of Alberta Community Health Committee (HREBA‐CHC).

### 2.3 Study cohort

The study cohort were adults (≥18 years of age) identified with clinical ASCVD, as defined by algorithms using International Classification of Diseases, Ninth Revision, Clinical Modification (ICD‐9‐CM) or International Statistical Classification of Diseases and Related Health Problems, 10th Revision, Canada (ICD‐10‐CA) diagnostic code for ASCVD. These conditions included: AMI, coronary atherosclerosis/historical MI, cerebrovascular disease including stroke, transient ischemic attack, unstable angina, peripheral arterial disease, and patients with percutaneous coronary intervention or coronary artery bypass graft surgery in any diagnosis position in the DAD, NACRS, or Practitioner Claims datasets. Details of the ASCVD diagnostic codes and algorithms are provided in Table S1. To estimate period prevalence, patients meeting the ASCVD algorithm criteria were identified between fiscal years 2011/2012 and 2015/2016 (April 1, 2011 and March 31, 2016).

For examining the 1‐year rate of subsequent MACE outcomes, cases were ascertained between April 1, 2012 and March 31, 2016, and each case was required to have a 1‐year follow‐up period. The index date for inclusion was the first or second ASCVD diagnostic code date (depending on the ASCVD condition). The absence of any ASCVD diagnostic code in a 2‐year lookback period was required for inclusion into these analyses, which served as a proxy to identify incident ASCVD cases. A subgroup of patients with AMI was derived from the overall ASCVD cohort, based on having an AMI event as the index ASCVD diagnostic event. Since this AMI subgroup was derived based on the index ASCVD event within the study period, the subgroup does not represent the total AMI population in Alberta.

### 2.4 Study variables

Demographic characteristics included: age, sex, and Alberta geographic health zone at the ASCVD diagnosis index date. Clinical characteristics included: prescription dispense for LLT (defined as statins, statin intensities, adjunctive ezetimibe and other LLT [Table S2]) within 6 months post‐ASCVD index date, and Charlson Comorbidity Index (CCI) scores derived within 1‐year of the ASCVD index date.

MACE outcomes were extracted from the DAD, NACRS, or vital statistics datasets based on ICD‐10‐CA/ICD‐9‐CM codes as the primary diagnosis 30 days after and within 1‐year following ASCVD diagnosis; and examined as two composite outcomes: primary MACE (CV death, AMI, stroke, hospitalization for unstable angina [only extracted from the DAD], and coronary revascularization), and secondary MACE (CV death, AMI, and stroke).^23^ Individual MACE outcomes were also examined (Table S3). Subsequent MACE outcomes occurring within the first 30‐days of the ASCVD diagnosis index date were excluded to remove any follow‐up records attributed to the initial ASCVD diagnostic event.

### 2.5 Statistical methods

The 5‐year period prevalence was calculated by dividing the number of patients identified with clinical ASCVD in Alberta between April 1, 2011 and March 31, 2016 (numerator) with the estimated total population of Alberta in 2015 (denominator—the most recent year in the prevalence calculation) using publicly available population estimates from Statistics Canada.^24^ Period prevalence estimates were stratified by age categories (20–49, 50–64, 65–74, 75+ years) and sex (female, male).

Demographic and clinical characteristics were reported descriptively for the overall ASCVD population and for the subgroup of patients with an AMI as their index event. The 1‐year incidence rates (IRs) and corresponding 95% confidence intervals (CIs) of subsequent MACE outcomes were also calculated (events/100 person‐years). All statistical analyses presented here were performed in SAS® version 9.4.

## 3 RESULTS

### 3.1 ASCVD period prevalence

**FIGURE 1:**
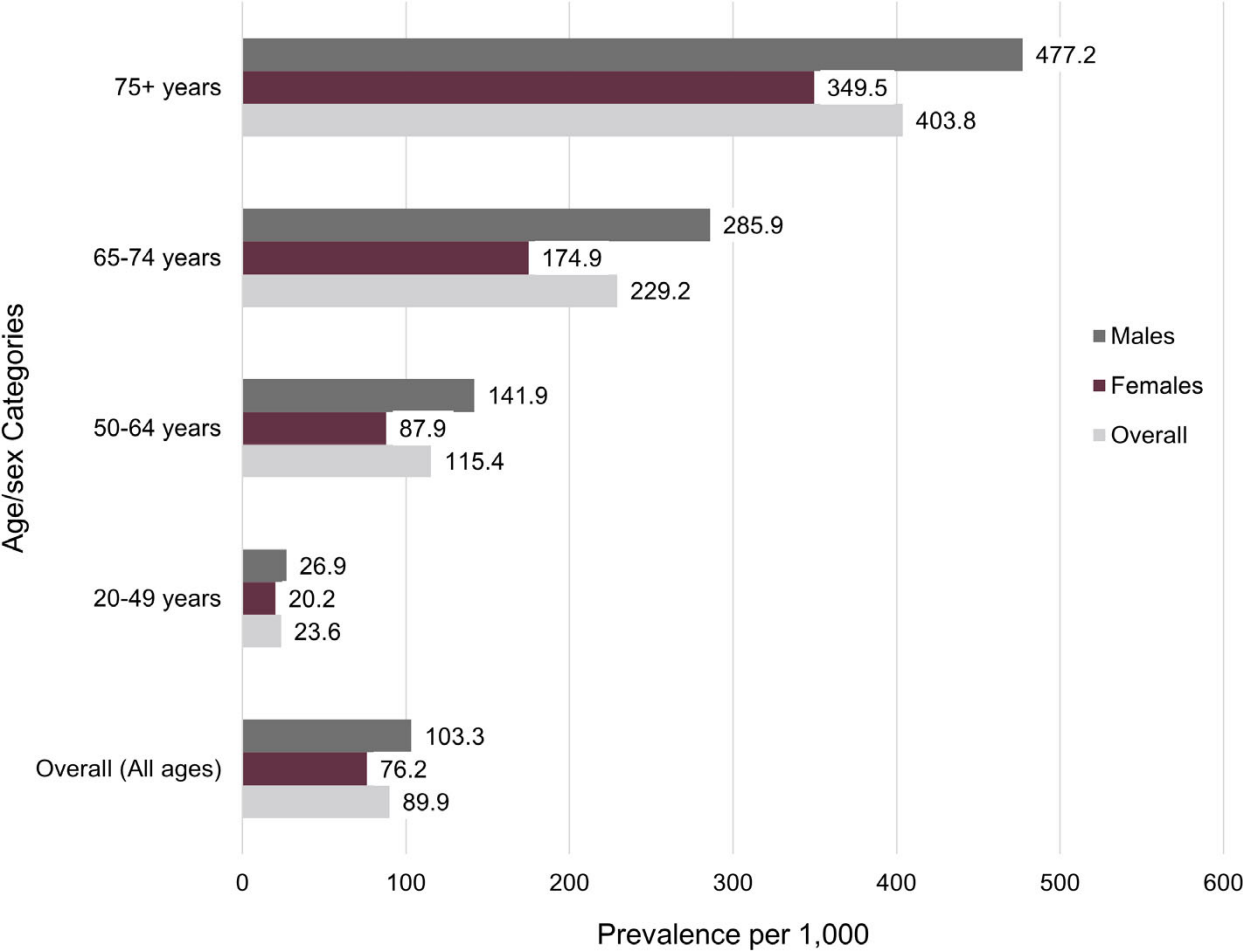
ASCVD period prevalence (per 1000 persons) in Alberta between fiscal years 2011/2012 and 2015/2016 (April 1, 2011 to March 31, 2016), by age and sex

The 5‐year period prevalence of ASCVD in Alberta was 89.9 per 1000 persons (Figure 1). The prevalence of ASCVD was higher in males than females (103.3 vs. 76.2 per 1000 persons) and increased with age. The highest prevalence was observed among males over the age of 75 years (477.2 per 1000 persons).

### 3.2 Study cohort and characteristics

A total of 198 573 patients met the ASCVD study cohort definition between April 1, 2012, and March 31, 2016, from the health system data in Alberta, Canada. Of those, 9465 patients were categorized as having an AMI as their index event.

The mean (standard deviation [SD]) age of the ASCVD study cohort was 63.9 (15.6) years (Table 1). Approximately half of the study cohort (49.5%) were ≥ 65 years old at their index date, 56.6% were male, and most patients (64.8%) had a CCI score of ≥1. Just under half of the study cohort (47.5%; *n* = 94 252) received statin therapy within the first 6 months following ASCVD index date; among those, almost all were prescribed moderate‐ or high‐intensity statins (96.9%). In the subgroup analysis, patients with an AMI index event were slightly older (mean age [SD] of 67.9 [15.4] years), had a higher proportion of patients with a CCI score of ≥1 (100%), and a greater proportion receiving statin therapy within 6 months of index (69.3%), relative to the overall ASCVD cohort.

**TABLE 1:** Patient characteristics of patients with ASCVD overall and the subgroup of patients with an index AMI event

Characteristics	ASCVD cohort, *n* = 198 573	Index AMI event subgroup, *n* = 9465
Age, mean (SD)	63.9 (15.6)	67.9 (15.4)
Age, *n* (%)		
<55	53 321 (26.9)	1956 (20.7)
55 to <65	47 000 (23.7)	2179 (23.0)
≥65	98 252 (49.5)	5330 (56.3)
Sex, *n* (%)		
Female	86 136 (43.4)	3608 (38.1)
Male	112 437 (56.6)	5857 (61.9)
Alberta geographic health zone, *n* (%)		
Calgary zone	60 995 (30.7)	2403 (25.4)
Central zone	23 002 (11.6)	1862 (19.7)
Edmonton zone	80 216 (40.4)	2849 (30.1)
North zone	20 293 (10.2)	1465 (15.5)
South zone	14 067 (7.1)	886 (9.4)
CCI score, *n* (%)		
0	69 953 (35.2)	0 (0.0)
1–2	76 287 (38.4)	4113 (43.5)
3+	52 333 (26.4)	5352 (56.5)
Received statin treatment, *n* (%)		
No	104 321 (52.5)	2910 (30.7)
Yes	94 252 (47.5)	6555 (69.3)
Statin treatment intensity, *n* (%)		
Low intensity	2952 (3.1)	85 (1.3)
Moderate intensity	50 476 (53.6)	1507 (23.0)
High intensity	40 824 (43.3)	4963 (75.7)
Received adjunctive ezetimibe, *n* (%)		
No	193 381 (97.4)	9316 (98.4)
Yes	5192 (2.6)	149 (1.6)
Received other LLT, *n* (%)		
No	189 475 (95.4)	9221 (97.4)
Yes	9098 (4.6)	244 (2.6)

Abbreviations: AMI, acute myocardial infarction; ASCVD, atherosclerotic cardiovascular disease; CCI, Charlson Comorbidity Index; LLT, lipid‐lowering therapy; SD, standard deviation. Other LLT included, fibrates, bile acid sequestrants, nicotinic acid and derivatives, and other esters/acids.

### 3.3 Subsequent MACE outcome rates

Within the overall ASCVD cohort, the proportion of patients with a subsequent primary and secondary MACE outcome 30 days after and within 1‐year following ASCVD diagnosis were 5.7% and 3.4%, respectively, and the most common individual MACE outcome was coronary revascularization (2.6%) (Table 2). The 1‐year IRs for subsequent primary and secondary MACE outcomes were 6.15 (95% CI: 6.03–6.26) and 3.67 (95% CI: 3.58–3.75) per 100 person‐years, respectively. Among the individual MACE outcomes following the ASCVD index date, coronary revascularization had the highest IR at 2.80 (95% CI: 2.72–2.88) per 100 person‐years, followed by CV death at 1.59 (95% CI: 1.54–1.65) per 100 person‐years, and AMI at 1.33 (95% CI: 1.28–1.39) per 100 person‐years, while hospitalization for unstable angina was the least common (IR: 0.48, 95% CI: 0.45–0.51) per 100 person‐years.

**TABLE 2:** One‐year subsequent MACE outcome incidence rates/100 person‐years in patients with ASCVD overall and the subgroup of patients with an index AMI event

Subsequent MACE outcomes after initial ASCVD/AMI event	ASCVD cohort, *n* = 198 573		Index AMI event subgroup, *n* = 9465	
	MACE outcome, *n* (%)	One‐year MACE, IR (95% CI)^a^	MACE outcome, *n* (%)	One‐year MACE, IR (95% CI)^a^
Primary MACE^b^	11 239 (5.7)	6.15 (6.03–6.26)	1024 (10.8)	14.30 (13.45–15.20)
Secondary MACE^c^	6812 (3.4)	3.67 (3.58–3.75)	749 (7.9)	10.21 (9.50–10.96)
Individual MACE^d^				
CV death	2993 (1.5)	1.59 (1.54–1.65)	275 (2.9)	3.60 (3.20–4.05)
AMI	2491 (1.3)	1.33 (1.28–1.39)	463 (4.9)	6.29 (5.74–6.89)
Stroke	1885 (0.9)	1.01 (0.96–1.05)	72 (0.8)	0.95 (0.75–1.19)
Hospitalization for unstable angina	892 (0.4)	0.48 (0.45–0.51)	84 (0.9)	1.11 (0.89–1.37)
Coronary revascularization	5169 (2.6)	2.80 (2.72–2.88)	351 (3.7)	4.72 (4.25–5.24)

Abbreviations: AMI, acute myocardial infarction; ASCVD, atherosclerotic cardiovascular disease; CI, confidence interval; CV, cardiovascular; IR, incident rate; MACE, major adverse cardiovascular event; MI, myocardial infarction.

^a^ Incidence rates are calculated based on the person‐time contribution in the 30 days following and within 1 year of the ASCVD index event.

^b^ Primary MACE outcomes (extracted from DAD/NACRS) were defined as: CV death, acute myocardial infarction, ischemic stroke, hospitalization for unstable angina, or coronary revascularization.

^c^ Secondary MACE outcomes (extracted from DAD/NACRS) were defined as: CV death, acute myocardial infarction, or ischemic stroke.

^d^ Individual MACE outcomes are mutually exclusive, and for the primary and secondary composite outcomes, only the first event was counted towards the incident rate.

For the subset of patients with an AMI index event, the proportion of a subsequent primary and secondary MACE outcome 30 days after and within 1‐year following AMI diagnosis were 10.8% and 7.9%, respectively, with a subsequent AMI being the most common individual MACE outcome at 4.9%. These patients had 1‐year IRs for subsequent primary and secondary MACE outcomes of 14.30 (95% CI: 13.45–15.20) and 10.21 (95% CI: 9.50–10.96) per 100 person‐years, respectively. When examining individual MACE outcomes following an index AMI event, subsequent AMI was the most common MACE outcome (IR: 6.29, 95% CI: 5.74–6.89 per 100 person‐years), whereas stroke was the least common MACE outcome (IR: 0.95, 95% CI: 0.75–1.19 per 100 person‐years).

## 4 DISCUSSION

This study examined real‐world data from the health system of Alberta, Canada to describe the prevalence of ASCVD, as well as the rate of subsequent MACE outcomes following an initial ASCVD event and within a subgroup of patients with an index AMI event. The 5‐year period prevalence of ASCVD in Alberta was 89.9 per 1000 persons, or 8.99%, which aligns with previously published data from Statistics Canada reporting the 10‐year risk of a CV disease event to be 8.9%.^4^ Further, our study followed similar trends to the global burden of ASCVD, regarding higher rates of ASCVD prevalence among males and those with increasing age.^1^

In the overall ASCVD incident cohort, the 1‐year rate for subsequent primary and secondary MACE outcomes were 6.15 and 3.67 per 100 person‐years, respectively, with coronary revascularization as the most common MACE outcome. When examining the subgroup of patients with an index AMI event, 1‐year rates for subsequent primary and secondary MACE outcomes were much higher at 14.30 and 10.21 per 100 person‐years, respectively, relative to the overall ASCVD cohort. Further, among patients with an index AMI event, the rate for subsequent AMI events was the highest among all the individual MACE outcomes.

Other studies in the literature have also reported 1‐year subsequent MACE outcomes with notable similarities and differences. First, a pooled patient‐level analysis including four major antithrombotic therapy trials post‐ACS presentation (including AMI)^16^ reported a rate of secondary MACE outcomes (MI, stroke, or CV death) to be 9.2%, with subsequent MI being the most common (5.8%), followed by CV death (2.4%), and stroke (1.0%). Interestingly, these results follow similar trends to the AMI subgroup data in our study, with a secondary MACE outcome rate (defined as MI, stroke, or CV death) of 10.21 per 100 person‐years, and a subsequent AMI rate of 6.29 per 100 person‐years.

Further, our results fall within the range of subsequent MACE outcome rates reported in a comprehensive systematic review, which included observational studies examining patients with a history of ASCVD, high LDL‐C levels, or receiving LLTs. This review reported subsequent MACE outcomes rates ranging from 2.6 to 21.1 per 100 person‐years.^21^ Another Canadian study using medical services and hospitalization data from Manitoba reported 79.5% of patients identified with MI between 2006 and 2010 survived at least 1 year without a subsequent MI or stroke.^25^ In comparison, our study reported 95.1% and 99.2% of patients in the AMI subgroup did not experience a subsequent AMI, or stroke within 1‐year of follow‐up. It is important to note that the Manitoba study was a prevalent population‐based sample, whereas the current study investigated a subset of patients with AMI as their index event, thus individuals with prevalent AMI were omitted. Therefore, differences in results may also be attributed to improvements in MI care, since the Manitoba study included patients identified from 2006 to 2010 relative to our study which identified patients between 2012 and 2016. A study using United Status IBM MarketScan data observed a lower 1‐year MACE outcome rate post‐ASCVD (including MI, stroke, and CV death) of 2.12 events per 100 person‐years relative to the current study at 3.67.^26^ This study only identified outcomes based on hospitalizations, whereas this study captured hospitalizations, ambulatory care visits, and vital statistics to define MACE outcomes in Alberta. These comparisons provide evidence that the current results may be generalizable to a broader Canadian population, especially given that the prevalence of ASCVD is similar to the overall Canadian estimate.^4^

Given the substantial burden of ASCVD and AMI across populations, secondary prevention efforts are essential for patient management. The most studied modifiable risk factor of CV disease is low‐density lipoprotein cholesterol (LDL‐C).^27^ Canadian guidelines recommend statins for all patients with established ASCVD, additionally, ezetimibe and PCSK9 inhibitors are recommended to lower LDL‐C,^28^ and decrease the risk of subsequent CV events in ASCVD patients above the LDL‐C threshold of 1.8 mmol/L.^18^, ^29^, ^30^, ^31^ However, despite an emphasis on secondary prevention of CV events with lipid‐lowering therapies, there remains a high residual risk for recurrent MACE outcomes and a substantial overall ASCVD burden.^19^ Our study found that only 47.5% of patients with ASCVD and 69.3% of patients with an AMI event received statin therapy within 6 months after their events. Recent international guidelines have recognized the importance of additional LLTs to ensure patients with ASCVD achieve guideline‐recommended LDL‐C goal levels^18^, ^28^, ^32^ and further reduce the risk of subsequent events. Additionally, recent guidelines have lowered the recommended LDL‐C goals or thresholds to intensify treatment for very high‐risk ASCVD patients.^28^ Further real‐world evidence to determine the impact of additional LLTs in addition to statins, as well as the resulting achievement of lower LDL‐C levels, on subsequent MACE outcomes are warranted.

Finally, this study should be interpreted in the context of several methodological limitations. First, the data used in this study were collected for billing, monitoring, and hospital administrative purposes, not for research. As a result, a diagnosis based on ICD codes alone does not confirm the presence of the disease. Second, important patient and clinical characteristics, which represent important ASCVD risk factors, are not captured in administrative data, such as modifiable lifestyle factors, behaviors, and ethnicity. Third, the subgroup of patients with an index AMI event was derived from the overall ASCVD cohort, based on the first ASCVD diagnostic event in the case ascertainment period (i.e., ASCVD index date). Therefore, this subgroup of patients is smaller and not representative of the overall AMI population in Alberta. Furthermore, this ASCVD cohort included patients indexed between 2012 and 2016, and treatment recommendations at the time from the 2012 CCS guidelines^33^ did not have recommendations for non‐statin therapy. Additionally, proprotein convertase subtilisin/kexin 9 inhibitors (PCSK9is) were not approved in Canada until 2015. Upon the publication of the 2016 guidelines, ezetimibe and PCSK9is were newly included in the Canadian treatment guidelines as add‐ons to statin therapy to get patients below LDL‐C goal. The introduction, uptake, and potential benefits of adjunctive ezetimibe and PCSK9i,^34^, ^35^ would not be captured in this study, even though they are now more widely used. While temporal treatment patterns were not the objective of this study, treatment outcomes from an Alberta‐based sample were evaluated in our previous publication and could be expanded upon in future studies.^36^ Lastly, while subsequent MACE outcomes that occurred within 30 days after the initial ASCVD index date were excluded, other events occurring past 30 days may be incorrectly categorized as a subsequent MACE outcome. This limitation may be pertinent when interpreting the coronary revascularization results, given how common coronary revascularization procedures are in clinical practice.^37^

In summary, by linking several comprehensive health administrative datasets, this study described the prevalence of ASCVD and the burden of subsequent MACE outcomes after ASCVD in Alberta. Province‐wide hospitalizations, ambulatory care visits, physician visits, and up‐to‐date cause of death data (e.g., CV death) were captured for all Alberta residents who met inclusion criteria, a strength in this study. Also, both composite and individual MACE outcomes were examined, providing evidence on the burden of subsequent MACE outcomes, and highlighting the need for secondary prevention strategies. Future research distinguishing initial from recurrent MACE outcomes, through prospective data collection, would help to confirm our results and further understand the long‐term burden of ASCVD in Canada. Furthermore, investigating other high‐risk ASCVD subgroups, such as those with peripheral artery disease, comorbid diabetes or metabolic syndrome, or familial hypercholesterolemia,^28^ would provide additional context for understanding the burden of ASCVD, and which subpopulations may be most affected. Finally, investigations examining patient management strategies (i.e., reducing cardiometabolic risk factors) to reduce initial ASCVD diagnoses and subsequent MACE outcomes after ASCVD are also needed.

## 5 CONCLUSIONS

Overall, results from the current study reveal that the prevalence of ASCVD in Alberta and the risk of subsequent MACE outcomes after an initial ASCVD event are substantial. Further, patients with an AMI event have considerably higher 1‐year MACE outcome rates, relative to the overall ASCVD cohort. These results suggest that more rigorous management of ASCVD, and particularly AMI, could be investigated further to improve health outcomes among patients with ASCVD in Alberta.

## CONFLICT OF INTEREST

GC is a consultant for Medlior which received funding for the study from Amgen. MSF and TC are employed by Medlior, which received funding for the study from Amgen. LP, RR, EM, and SC are employed by Amgen who funded this study and hold Amgen stock. TA received research funding from Amgen, Novartis and Dal‐cor for being a local Principal Investigator for randomized clinical trials.

## AUTHOR CONTRIBUTIONS

Guanmin Chen, Megan S. Farris, Tara Cowling, Lionel Pinto, Raina M. Rogoza, and Erin MacKinnon collaborated on the study design of the project. Guanmin Chen, Megan S. Farris, and Tara Cowling were responsible for requesting, analyzing, and reporting data. All authors developed, read, and approved the final manuscript.

## ETHICS STATEMENT

The study complies with the Declaration of Helsinki and was approved by the Health Research Ethics Board of Alberta Community Health Committee (HREBA‐CHC).

## Supporting information


**Appendix S1**: Supporting information.

## Data Availability

Data from this study are not publicly available and cannot be shared due to privacy reasons and ethical restrictions, as per the research agreement with Alberta Health.
