# Supplementary material for: Prevalence of atherosclerotic cardiovascular disease and subsequent major adverse cardiovascular events in Alberta, Canada: A real‐world evidence study
Source: Clin Cardiol. 2021 Sep 29;44(11):1613–20. doi: 10.1002/clc.23732 (PMC8571560; doi:10.1002/clc.23732)
Supplement: Supplementary file 1 — Appendix S1: Supporting information. [file CLC-44-1613-s001.docx]

Supplementary Table 1. ICD-9-CM/ICD-10-CA codes for the ASCVD cohort in Alberta

| **ASCVD conditions^a^** | **ICD-9-CM** | **ICD-10-CA** | **Definition Algorithm during the cohort year** |
| --- | --- | --- | --- |
| AMI | 410 | I21, I22 | 1 IP (Inpatient only), any position (discharge date as index date) |
| Unstable angina | 411, 413 | I20 | 1 IP (inpatient only), any position; OR 1 Physician claim, any position; OR 1 ED visit, any position (all discharge date =index date); earliest as index date |
| Cerebrovascular/stroke | 430-434, 436-438, 3623 | I60-I65, I67, I69, H341 | 1 IP (discharge date); OR 2 physician claims/ED visits at least 30 days apart; any position (second date=index date) earliest as index date |
| Transient ischemic attack | 435 | G450-G453, G458-G459, H340 | 1 IP, any position (discharge date=index date); OR 2 physician claims/ED visits 30 days apart, any position (second date =index date); earliest as index date |
| Coronary atherosclerosis/historical MI | 412, 414 | I25 | 1 IP (discharge date, any position); OR 2 physician claims/ED visits (second date=index date), claims at least 30 days apart; any position; earliest as index date. |
| Peripheral arterial disease | 4439, 4402 | I739, I702, I792 | 1 IP (any position, discharge date); OR 2 physician claims/ED visits 30 days apart (second date=index date); earliest as index date. |
| Percutaneous coronary intervention | CCP codes: 4802, 4803 | Canadian Classification of Health Intervention codes: 1IJ50, 1IJ57GQ, 1IJ54 | 1 IP or 1 ED CCP/ Canadian Classification of Health Intervention code (Procedure date if available; otherwise discharge date); earliest date as index date |
| Coronary artery bypass graft surgery | CCP codes: 481 | Canadian Classification of Health Intervention codes: 1IJ76 | 1 IP or 1 ED CCP/ Canadian Classification of Health Intervention code (Procedure date if available; otherwise discharge date); earliest date as index date |

Abbreviations: AMI: acute myocardial infarction; ASCVD; atherosclerotic cardiovascular disease; CCP: Canadian Classification of Diagnostic, Therapeutic, and Surgical Procedures; ED: emergency department; ICD 9-CM: International Classification of Diseases, Ninth Revision, Clinical Modification; ICD-10-CA: International Statistical Classification of Diseases and Related Health Problems, 10th Revision, Canada; IP: inpatient; MI: myocardial infarction.

^a^ The ASCVD cohort included all of the above conditions listed.

Supplementary Table 2. ICD-10-CA codes for the MACE outcomes

| **MACE outcomes** | **ICD-10-CA** | **Definition Algorithm during the cohort year** |
| --- | --- | --- |
| CV death | Ischemic heart disease death: I21-I25; cerebrovascular disease death: I60-I69; circulatory disease death: I00-I99 | 1 IP or 1 ED death with corresponding ICD-10-CA code; OR vital statistics record with corresponding ICD-9-CM code |
| AMI | I21, I22 | 1 IP or 1 ED visit |
| Stroke | I63, I64, H341 | 1 IP or 1 ED visit |
| Hospitalization for unstable angina | I200 | 1 IP (inpatient only) |
| Coronary revascularization (PCI and CABG surgery) | 1IJ50, 1IJ57GQ, 1IJ54, 1IJ76 | 1 IP or 1 ED visit |

Abbreviations: AMI: acute myocardial infarction; CABG: coronary artery bypass graft surgery; CV: cardiovascular; ED: emergency department; ICD 9-CM: International Classification of Diseases, Ninth Revision, Clinical Modification; ICD-10-CA: International Statistical Classification of Diseases and Related Health Problems, 10th Revision, Canada; IP: inpatient; MACE: major adverse cardiovascular events; PCI: percutaneous coronary intervention.

Supplementary Table 3. Lipid-lowering therapy ATC classification codes

| **Classification** | **ATC code** | **Drug names** |
| --- | --- | --- |
| Statins, statins with other combinations | C10AA01, C10AA02, C10AA03, C10AA04, C10AA05, C10AA07, C10BX03 | Simvastatin, lovastatin, pravastatin, fluvastatin, atorvastatin, rosuvastatin, atorvastatin and amlodipine |
| Fibrates | C10AB02, C10AB04, C10AB05 | Bezafibrate, gemfibrozil, fenofibrate |
| Bile acid sequestrants | C10AC01, C10AC02, C10AC04 | Colestyramine, colestipol, colesevelam |
| Nicotinic acid | C10AD02 | Nicotinic acid |
| Other lipid modifying agents | C10AX06, C10AX09, C10AX13 | Omega-3-triglycerides included other esters and acids, ezetimibe, evolocumab |

Abbreviations: ATC: anatomical therapeutic chemical

Note: Medication types were presented separately in results tables, permitting sample size (cells representing n<5 individuals are suppressed to reduce the risk of re-identification of individuals).
